# Copper Exposure Induces Epithelial-Mesenchymal Transition-Related Fibrotic Change via Autophagy and Increase Risk of Lung Fibrosis in Human

**DOI:** 10.3390/antiox12020532

**Published:** 2023-02-20

**Authors:** Hsin-Ying Clair Chiou, Chih-Wen Wang, Szu-Chia Chen, Mei-Lan Tsai, Ming-Hong Lin, Chih-Hsing Hung, Chao-Hung Kuo

**Affiliations:** 1 Teaching and Research Center, Kaohsiung Municipal Siaogang Hospital, Kaohsiung Medical University, Kaohsiung 807, Taiwan; 2 Kaohsiung Medical University Hospital, Kaohsiung Medical University, Kaohsiung 807, Taiwan; 3 Department of Applied Chemistry, National Chi Nan University, Nantou 540, Taiwan; 4 Division of Hepatobiliary, Department of Internal Medicine, Kaohsiung Medical University Hospital, Kaohsiung Medical University, Kaohsiung 807, Taiwan; 5 Department of Internal Medicine, Kaohsiung Municipal Siaogang Hospital, Kaohsiung Medical University, Kaohsiung 807, Taiwan; 6 Research Center for Environmental Medicine, Kaohsiung Medical University, Kaohsiung 807, Taiwan; 7 Division of Nephrology, Department of Internal Medicine, Kaohsiung Medical University Hospital, Kaohsiung Medical University, Kaohsiung 807, Taiwan; 8 Graduate Institute of Medicine, College of Medicine, Kaohsiung Medical University, Kaohsiung 807, Taiwan; 9 Department of Pediatrics, Faculty of Pediatrics, College of Medicine, Kaohsiung Medical University, Kaohsiung 807, Taiwan; 10 Department of Microbiology and Immunology, School of Medicine, College of Medicine, Kaohsiung Medical University, Kaohsiung 807, Taiwan; 11 Master of Science Program in Tropical Medicine, College of Medicine, Kaohsiung Medical University, Kaohsiung 807, Taiwan; 12 Department of Medical Research, Kaohsiung Medical University Hospital, Kaohsiung 807, Taiwan; 13 Department of Pediatrics, Kaohsiung Medical University Hospital, Kaohsiung Medical University, Kaohsiung 807, Taiwan; 14 Department of Pediatrics, Kaohsiung Municipal Siaogang Hospital, Kaohsiung Medical University, Kaohsiung 807, Taiwan; 15 Division of Gastroenterology, Department of Internal Medicine, Kaohsiung Medical University Hospital, Kaohsiung Medical University, Kaohsiung 807, Taiwan

**Keywords:** copper, pulmonary fibrosis, epithelial-mesenchymal transition, flavonoid, autophagy

## Abstract

Copper is an essential trace element involved in several vital biological processes of the human body. However, excess exposure to copper caused by occupational hazards and environmental contamination, such as food, water, and air, damages human health. In this study, in vitro cell culture model and epidemiologic studies were conducted to evaluate the effect of copper on lung fibrosis. In vitro, treatment of CuSO_4_ in lung epithelial cells at 100 μM consistently decreases cell viability in alveolar type (A549) and human bronchial epithelial (HBE) cells. CuSO_4_ promotes epithelial-mesenchymal transition (EMT) as shown by increased cell migration and increased EMT marker and fibrotic gene expressions. Besides, CuSO_4_ induced cell autophagy, with an increased LC3, PINK, and decreased p62 expression. Inhibition of ROS by N-acetylcysteine reversed the CuSO_4_-induced PINK1, LC3, and Snail expressions. Inhibition of autophagy by chloroquine reverses the CuSO_4_-induced EMT changes. Nature flavonoids, especially kaempferol, and fustin, were shown to inhibit Copper-induced EMT. In humans, a unit increase in urinary copper concentration was significantly associated with an increased risk of lung fibrotic changes (odds ratio [OR] = 1.17, 95% confidence interval [CI] = 1.01–1.36, *p* = 0.038). These results indicated that Copper is a risk factor for lung fibrosis through activation of the ROS-autophagy-EMT pathway, which can be reversed by flavonoids.

## 1. Introduction

Copper is an essential trace element that acts as a cofactor of many enzymes involved in redox reactions and the electron transport system [1,2]. The cellular concentration of copper was balanced by absorption (copper transporter (Ctr)-1, 2), and secretion (ATP7A and ATP7B) by copper-binding proteins. Over-accumulation of copper was known to result in several pathological conditions including Wilson’s disease, Menke’s disease (MD), hemolytic anemia, gastrointestinal bleeding, and diarrhea [3]. With the increasing use of copper in the manufacturing processes, such as semiconductor devices, copper pollution occurred through air and wastewater. Previous studies revealed that subjects with diabetes mellitus, alcohol consumption, betel quid chewing, or cigarette smoking had significantly higher urinary copper levels than those without [4,5,6]. Thus, copper toxicity is considered a rising issue for human health. Some studies show copper toxicity in the lung. People exposed to copper sulfate, which was used to combat mildew in vineyards, have a higher risk to develop granulomatous lung disease and progressive massive fibrosis [7]. In an experimental model, mice inhalation of copper nanoparticles caused pulmonary toxicity and lung fibrosis [8]. Since people can be exposed to copper pollutants through several routes, whether urinary copper concentration in humans is correlated with lung function impairment and lung fibrotic changes is still unknown. 

Airway epithelial cells, in addition to serving as the physical barrier to foreign insults, are critical in orchestrating the fibrotic process. Epithelial cells undergo a transition into the mesenchymal type of cells upon stimuli, named epithelial-mesenchymal transition (EMT) process. The cells lose the epithelial phenotype and acquire the mesenchymal phenotype, including increased cell mobility, a reorganized cytoskeleton, and increased invasiveness and production of the extracellular matrix. The transformed mesenchymal cell contributed a significant proportion of activated fibroblast, and its release of pro-fibrogenic factors would further activate other fibroblast and excess deposition of extracellular matrix proteins, which is a characteristic of tissue fibrosis. EMT is a reversible process. EMT is considered the main culprit, and the targeting of EMT was considered a therapeutic target for fibrotic disorders [9,10,11,12]. The application of natural compounds for reverse EMT, such as diosgenin and sulforane, was effective in attenuating lung fibrosis in mice studies [13]. 

Flavonoids are a group of natural products, including flavanones, flavanonols, isoflavonoids, flavans, and so on. They are abundant in vegetables and fruits and have broad biological activities such as anti-inflammatory, anti-oxidative, and anti-tumor. The administration of apigenin, which is a flavanone, inhibits the metastasis of liver cancer and reduces mesenchymal protein expression [14]. In an idiopathic pulmonary fibrosis rodent model, lung fibrosis was attenuated by daily ingestion of apigenin [15]. Besides, the anti-oxidant cellular responses of flavonoids were through modulation of mitochondria biogenesis, autophagy, and fission and fusion for cytoprotection [16]. 

The copper that is excreted mainly in the feces as bile is the major excretory route, however, 0.5–3.0% of daily copper intake is excreted in the urine. In human biomon-itoring, copper concentrations in both blood and urine were assessed [17,18]. Blood copper levels were influenced by the production of ceruloplasmin; however, urinary copper levels were not. In this study, we aimed to investigate the association of urinary copper levels with lung fibrotic changes in individuals without cigarette smoking. We also performed the evaluation of the therapeutic effects and mechanisms of several flavonoids on copper-induced lung epithelial damage.

## 2. Materials and Methods

### 2.1. Cell Culture, Drugs, and Reagents

Normal human bronchial epithelial cells (NHBE, Lonza, Basel, Switzerland) were purchased from Lonza (Cat. No. CC-2450) and were maintained in Keratinocyte SFM basal medium supplement with 5 μg/L human recombinant epithelial growth factor (EGF), 50 mg/L Bovine Pituitary Extract (BPE), 5 mg/L insulin and 25 nM hydrocortisol. Cells were passaged every 3 days and plated for experiment treatment within 6 passages. Human alveolar type II epithelia cells A549 cell was purchased from ATCC (Cat.No.CCL-185) and were maintained in DMEM medium supplement with 10% FBS, sodium pyruvate, and 1% penicillin/streptomycin. Cells were maintained at 37 °C in a 5% CO_2_ humidified atmosphere. CuSO_4_, chloroquine, N-acetylcysteine, fusin, apigenin, kaempferol, and genkwanin were purchased from Sigma-Aldrich (St. Louis, MO, USA). 

### 2.2. Western Blotting

For protein expression analysis after treatment, the cells were washed twice with ice-cold PBS, and lysed immediately with the addition of RIPA buffer containing protease inhibitor (cOmplete™, Mini, EDTA-free Protease Inhibitor Cocktail, Sigma-Aldrich, St. Louis, MO, USA) and phosphatase inhibitor (PhosStop™, Sigma-Aldrich, St. Louis, MO, USA) directly into the culture plate. The protein lysate was harvested by centrifugation with 14,000 rpm at 4 °C for 15 min to pellet the cell debris. The protein concentration was determined using BCA Dual-Range BCA Protein Assay Kit (Visual Protein, Neihu, Taiwan). A total of 10 ug of protein lysate separated in 6~13% SDS-PAGE and transferred onto PVDF membranes (Millipore, Bedford, MA, USA). After blocking at 5% skim milk at room temperature for 1 h, the membrane was incubated with first antibody at 4 °C overnight. After three times washes with 0.1%TBST, the membrane was incubated with HRP-conjugated anti-rabbit IgG or anti-goat secondary antibody, respectively, (Jackson ImmunoResearch Laboratories, West Grove, PA, USA). The membrane was developed by reacting with chemiluminescence HRP substrate (Merck, Darmstadt, Germany) and the protein bands were visualized and captured using ChemiDoc-It 810 Imager (Ultra-Violet Products, Upland, CA, USA). The protein bands were quantified using Image J. Antibodies used are including N-Cadherin (GTX127345, Genetex, Hsinchu, Taiwan), E-Cadherin (GTX100443, Genetex, Hsinchu, Taiwan), GAPDH (GTX100118, GeneTex, Genetex, HsinChu), PINK1 (Cell signaling #6946, USA), LC3 (GTX 127375, GeneTex, Hsinchu, Taiwan), p62 (abcam), Snail (GTX 100754, Genetex, Hsinchu, Taiwan).

### 2.3. Wound Healing Assay

A549 cells were plated in a 12-well plate. Cells were pre-treated with inhibitors for 2 h followed by combined treatment with CuSO_4_ for another 24 h. Wounds were made with 200 μL tips, followed by PBS wash two times to remove the floating cells. The images were then captured at different time points as indicated using microscopy. The wound area was analyzed by Image J. The cell migration activity was expressed as GAP% which was calculated as [(wound area of indicated time point)/(wound area of 0 h)] × 100%.

### 2.4. RNA Extraction and Semi-Quantitative Real-Time PCR

A549 cells grown in 12-well plates were treated with 0, 1, 10, and 100 μM of CuSO_4_ for 48 h. Total RNA was isolated using GENEzol^TM^ TriRNA Pure Kit (Geneaid). The complementary DNA was synthesized by using SuperScript^TM^ III reverse transcriptase (ThermoFisher Scientific, MA, USA). The gene expressions were detected with Fast SYBR Green MasterMix (Applied Biosystems) using StepOne system (ABI). The gene accession and primers for real-time PCR analysis are: 

COL1A1: NM_000088, primer sequences: Forward: 5′-CCAGAAGAACTGG TACATCAGCA-3′ and Reverse: 5′-CGCCATACTCGAACTGGAATC-3′; MMP-7: NM_002423, Forward: 5′-TCGGAGGAGATGCTCACTTCGA-3′ and Reverse: 5′-GGATCAGAGGAATGTCCCATACC-3′; LOXL2: NM_002318, Forward: 5′-TGACTGCAAGCACACGGAGGAT-3′ and Reverse: 5′-TCCGAATGTCCTCCACCTGGAT-3′; N-Cadherin: NM_001792, Forward: 5′-CCTCCAGAGTTTACTGCCATGAC-3′ and Reverse: GTAG GATCTCCGCCACTGATTC.

### 2.5. Transwell Migration Assay

A549 cells were exposed to apigenin for 2 h and followed by CuSO_4,_ exposed for another 24 h. The cells were detached by trypsin (Millipore, Freehold, NJ, USA) and seeded onto the upper chamber of transwell with 8 μm pore size (BD Biosciences, Franklin Lakes, NJ, USA) in a density of 4 × 10^4^ cells in 200 uL of serum-free DMEM. The lower chamber was filled with complete culture medium with drugs. After 24 h incubation, the cells were fixed with methanol for 10 min at room temperature followed by staining with 0.2% crystal violet for 10 min at room temperature. The cells in the upper side of the chamber were removed by cotton swab. The migration cells were determined by counting the cell number from 5 randomly selected area. 

### 2.6. Immunofluorescence

Cells plated on coverslips were treated by CuSO_4_ at various times and doses. After treatment, the cells were fixed by 10% formalin at room temperature for 30 min. After washing three times by PBS, the cells were then permeabilized with ice-cold 100% methanol for 10 min. Followed by 3 times of PBS wash, the cells were incubated with LC3 antibody (1:200, Genetex, Hsinchu, Taiwan) at 4 °C overnight. The non-specific binding was washed by 0.02% PBST, followed by incubation with goat-anti-rabbit-Alexa488 (Jackson) at room temperature for 2 h. The cells were then washed with 0.02% PBST and incubated with Alexa647-Tomm20 (1:100) overnight. After PBS wash, the cells were mounted by mounting solution with DAPI. The cells were analyzed by laser confocal microscope (FV1000, OLYMPUS IX-81). 

### 2.7. Participant Recruitment 

We conducted a general population health survey and lung cancer screening in Dalinpu in 2016 and 2018. Dalinpu is located in Xiaogang District, Kaohsiung City, southern Taiwan and was surrounded by Linyuan and Linhai petrochemical complex and oil refinery parks. Participants who lived for more than two years in Dalinpu were recruited through advertisements. Self-reported questionnaires (history of diabetes mellitus, hypertension, hyperlipidemia, education levels, physical activity, and air purifier use) and anthropometric measurements (including body weight, height, smoking, and pneumonia or lung carcinoma history), urinary copper and creatinine and Low-dose computed tomography (LDCT) were performed for all participants. Physical activity was asked as “Do you undertake at least 150 min per week of moderate-intensity aerobic activity or 75 min per week of vigorous aerobic activity or an equivalent combination?” Subjects with lung carcinoma who underwent lung lobectomy, or who had pneumonia history (*n* = 23), and those with cigarette smoking history (*n* = 283) were excluded. We excluded subjects with urinary creatinine concentration < 30 mg/dL and creatinine concentration > 300 mg/dL as the WHO recommendation was to prevent too diluted or too concentrated urinary samples (*n* = 88) (WHO, 1996). In total, 1458 individuals were included in the analysis. The study protocol was approved by the Institutional Review Board of Kaohsiung Medical University Hospital (Number: KMUHIRB-G(II)-20190011). 

### 2.8. Urinary Copper Analysis, Blood Biochemistry Analysis, and LDCT Image Acquisition and Interpretation

The blood samples were diluted 10-fold with 9 mL of 1% (*v*/*v*) nitric acid (J.T. Baker Chemical Company, Phillipsburg, NJ, USA) in 15 mL polypropylene tubes. The concentrations of urinary copper were determined by coupled plasma-mass spectrometry (ICP-MS, 7700 Series; Agilent Technologies, Inc., Santa Clara, CA, USA). The quality control and method detection limit are based on the standard of the National Environmental Laboratory. 

White blood cell count (WBC) and eosinophil count were analyzed by using a hematology analyzer (XE-2100; Sysmex, Kobe, Japan). Serum aspartate aminotransferase (AST), alanine aminotransferase (ALT), cholesterol, triglycerides, low-density lipoprotein cholesterol (LDL-C), high-density lipoprotein cholesterol (HDL-C), fasting glucose, and HbA1c levels were determined using a chemistry system (Advia 1800; Siemens Healthcare GmbH, Erlangen, Germany). The serum total IgE level was determined using ImmunoCAP^®^ (Phadia, Uppsala, Sweden).

All scans were performed on thin-slice scanners (Toshiba Aquilion One 640 slice CT scanner, Japan) with 320 detectors from the lung apex to the base without contrast enhancement. Moreover, all scans were obtained using a low-dose regimen and the following machine settings: tube voltage, 120 kVp; tube current, 9 (15 mA/0.6 s) or 21 (35 mA/0.6 s) mA; pitch ratio, 1.5:1; and rotation time, 0.35 s. The effective radiation dose ranged from 0.3 to 0.8 mSv. Lung fibrotic changes included parenchymal bands of lung fibrotic pattern that were defined as the presence of curvilinear or linear densities, fine lines, or plate opacity in specific lobes on LDCT images. All images were clinically reviewed and reported by trained radiologists with 10–30 years of experience.

### 2.9. Statistical Analysis

Lung fibrotic change, gender, alcohol and betel consumption, diabetes mellitus, and hypertension were considered nominal variables. Fisher’s exact or a chi-square test was used to determine the differences between lung fibrotic change. The Mann–Whitney U test was used to examine the differences between lung fibrotic and non-lung fibrotic changes in terms of urinary copper and creatinine levels and blood biochemistry levels. Correlation between urinary copper, age, BMI, and serum biochemistry levels was analyzed by Spearman correlation analysis. We conducted a multiple logistic regression analysis to assess the odds ratio (OR) and 95% confidence interval (CI) for lung fibrotic change associated with urinary copper levels (continuous variable). We included urinary creatinine levels as a covariate in the regression model to account for dilution-dependent sample variation in urine concentrations [19]. Variables (1) associated with lung fibrotic changes, (2) associated with urinary copper levels, and (3) based on the literature were selected. AST and ALT are both significantly associated with urinary copper levels and we chose AST as confounding factors to prevent multicollinearity. All analyses were performed using SPSS software (version 22; IBM Corp., Armonk, NY, USA). Statistical significance was set at *p* < 0.05. In in vitro and in vivo studies, the statistical analysis was performed using GraphPad Prism. The statistical significances between the two groups were performed using Student’s *t*-test and one-way ANOVA with Dunnett post hoc multiple comparison. The data were expressed as mean + SEM and the *p*-value was set at 0.05. 

## 3. Results

### 3.1. CuSO_4_ Induced EMT of Human Lung Epithelial Cells

**Figure 1:**
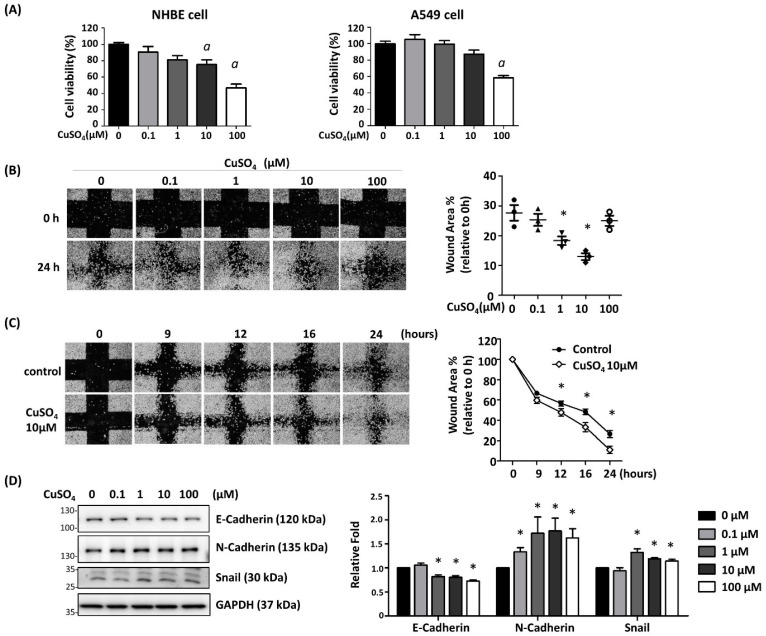
Dose and time effects of CuSO_4_ on cell viability and EMT. (**A**) HBE and A549 cells were treated with various doses of CuSO_4_ from 0.1~100 μM for 24 h, and the cell viability was determined by CCK-8 assay. *a*: *p* < 0.05 compared with the control group. (**B**) The dose effect of CuSO4 on A549 migration ability. A549 cells were treated with various doses of CuSO_4_ for 24 h, and wounds were made for wound healing assay. 12 h after treatment, the wound coverage was determined. (**C**) The wound coverage of CuSO_4_-treated cells at different time points. A549 were treated with 10 μM CuSO_4_ and the wound coverage was determined. (**C**) At different time points after the wound was made, the GAP% was determined. The wound area was measured and represented as GAP%. Mean + SEM. (**D**) A549 cells were treated with various doses of CuSO_4_ for 24 h. The protein expressions of EMT-related proteins were examined by Western blot. The quantitative results were shown in right panel. * *p* < 0.05. All experiments were performed three times and the representative image was shown.

To determine the effect of CuSO_4_ on lung epithelial cells, the dose effect of CuSO_4_ on cell viability was determined by CCK-8 assay. CuSO_4_ has significant toxicity at concentrations higher than 10 μM in human bronchial epithelial cells (HBE) and 100 μM in alveolar epithelial cells, A549 (Figure 1A). The effect of CuSO_4_ on cell migration was determined by wound healing assay. The results indicated that 1 μM, 10 μM, and 100 μM of CuSO_4_ significantly reduced the wound area (Figure 1B). The wound area (GAP%) was significantly reduced from 12 h after the wound was made (Figure 1C). These results indicated that Copper treatment promotes the cell migration of A549 cells. 

**Figure 2:**
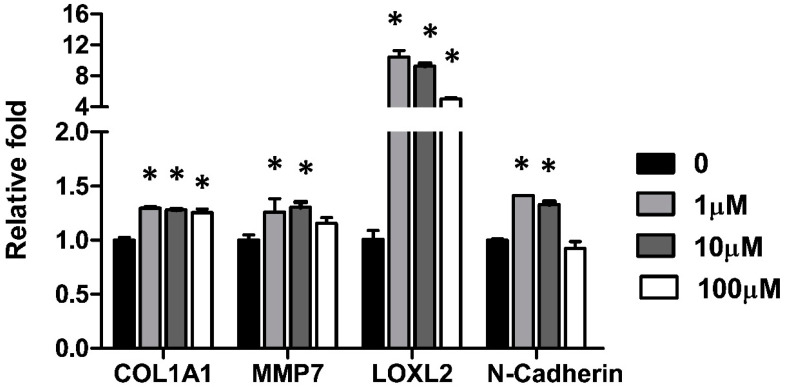
CuSO_4_ induced fibrosis-related gene expressions. A549 cells were treated with various doses of CuSO_4_ for 48 h and the gene expressions were analyzed by quantitative real-time PCR. The data were expressed as Mean ± SEM, * *p* < 0.05. The experiment was performed three times for quantification analysis.

The effect of CuSO_4_ on the epithelial-mesenchymal protein expressions was determined. A549 cells were treated with CuSO_4_ at doses from 0.1 μM to 100 μM for 24 h, and the protein expressions were determined by Western blot. The results show that CuSO_4_ increases expressions of mesenchymal proteins including N-Cadherin. The expression of the epithelial marker, E-Cadherin, was reduced by CuSO_4_ treatment at a dosage higher than 1 μM (Figure 1D). Moreover, several lung fibrotic marker expressions from cells treated with 1 uM for 48 h were examined by real-time PCR. The results show that COL1A1, MMP7, LOXL2, and N-Cadherin were upregulated by CuSO_4_ (Figure 2). These results demonstrated that CuSO_4_ treatment induced fibrogenic changes in the lung epithelial cells. 

### 3.2. CuSO_4_ Induced Mitophagy of Lung Epithelial Cells

**Figure 3:**
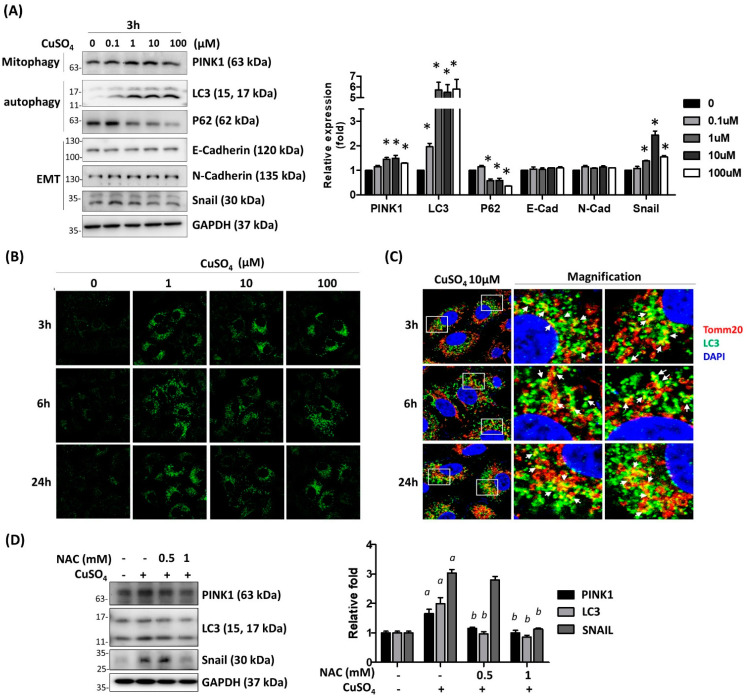
CuSO_4_ induced autophagy and mitophagy of lung epithelial cells. (**A**) A549 cells were treated with various doses of CuSO_4_ for 3 h. The protein expressions of mitophagy-, autophagy-, and EMT-related proteins were examined by Western blot. The quantitative results were shown in the right panel. Mean ± SEM were shown. *: *p* < 0.05. (**B**) A549 cells treated with various doses and time periods were fixed and applied to immunofluorescence staining using antibodies against LC3. (**C**) Co-localization of LC3 (green color) and Tomm20 (red color, indicating mitochondria) was shown in yellow color. (**D**) A549 cells were pretreated with 0.5, 1 mM NAC. After 2 h of treatment, the cells were treated in combination with 10 μM CuSO_4_ for 3 h, followed by Western blot analysis. *a*: *p* < 0.05 compared with the control group. *b*: *p* < 0.05 compared with CuSO_4_ only group. The quantitative results are shown in right panel. The experiments were performed three times for quantification analysis, and the representative images are shown.

To determine the effect of copper on mitophagy of the lung epithelial cells, A549 cells were treated with CuSO_4_ at doses from 0.1 μM to 100 μM. The expressions of mitophagy-specific markers PTEN-induced kinase 1 (PINK1), and autophagy-related proteins including LC3 and p62 were analyzed in cells treated with CuSO_4_ for 3 h by Western blot. The results show significant induction of PINK1 expressions upon 1 μM and 10 μM of CuSO_4_ treatment. Moreover, the expression of p62 was reduced by treatment of CuSO_4_ at a dose greater than 1 μM, accompanied by an increase in LC3 expressions (Figure 3A). In addition, the upregulation of LC3 was also demonstrated by immunofluorescence at 3, 6, and 24 h. The results indicated that the LC3 expression was induced by 1 μM, 10 μM, and 100 μM CuSO_4_ treatment at 3 h, and lasted for 24 h (Figure 3B). The intracellular localization of LC3 was partially co-localized with the mitochondria marker protein, Tomm20, as examined by confocal microscopy (Figure 3C). These results demonstrated that CuSO_4_ treatment activated the mitophagy of lung epithelial cells. 

Cupper exposure was known to enhance the production of reactive oxygen species (ROS) [17]. The inhibition of ROS by N-acetylcysteine inhibits the induction of PINK1, LC3, and Snail by CuSO_4_ dose-dependently (Figure 3D). This result indicated that the induction of autophagy by CuSO_4_ is mediated by ROS generation. 

### 3.3. Autophagy Inhibition Ameliorates the EMT Changes Induced by Copper

**Figure 4:**
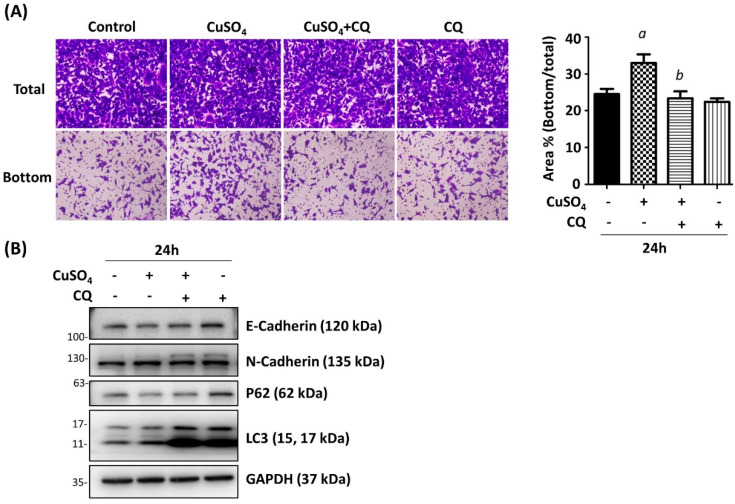
Inhibition of autophagy reversed CuSO_4_-induced cell migration. (**A**) A549 cells were pre-treated with chloroquine (CQ, 5 μM) for 2 h, followed by 10 μM of CuSO_4_ for another 24 h. *a*: *p* < 0.05 compared with the control group. *b*: *p* < 0.05 compared with CuSO_4_ only group. (**B**) The protein expressions were examined by Western blot. The quantitative results were shown in the right panel. Mean ± SEM.

To decipher the involvement of autophagy on copper-induced EMT changes, A549 cells were treated with chloroquine, which blocks the fusion of lysosome with autophagosome, 2 h before CuSO_4_ treatment. After 24 h of combined treatment, the cells were applied to a trans-well migration assay. Compared with the vehicle control, cells treated with CQ inhibit the cell migration induced by CuSO_4_ (Figure 4A). Moreover, the EMT marker expressions were determined by immunoblot. The results demonstrated that the autophagy was significantly blocked by CQ treatment as shown by the accumulated LC3II expression (Figure 4B). The decrease of E-Cadherin and the increase of N-Cadherin were reversed by the inhibition of autophagy (Figure 4B). These results indicate that the copper-induced EMT of A549 cells is mediated, at least partially, by the induction of autophagy. 

### 3.4. Flavonoid Is Effective to Ameliorate the Copper-Induce EMT Changes of Lung Epithelia Cells

**Figure 5:**
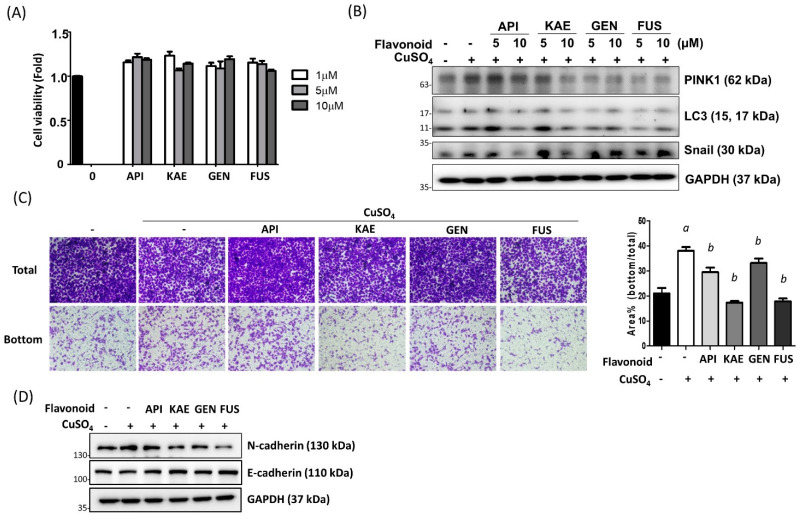
Flavonoid reversed CuSO_4_-induced EMT changes. (**A**) A549 cells were treated with 1 μM, 5 μM, and 10 μM of flavonoids (API: apigenin, KAE: kaempferol, GEN: genkwanin, FUS: fustin) for 24 h. After treatment, the cell viability was determined by CCK-8 assay. Mean ± SEM, (**B**) A549 cells were pre-treated with flavonoids for 2 h followed by combined treatment of 10 μM CuSO_4_ for 3 h. The protein expressions were determined by Western blot against PINK1, LC3, and Snail. GAPDH served as internal control. (**C**) A549 cells were pretreated with various flavonoids (10 μM) for 2 h followed by combined treatment with CuSO_4_ for 24 h. After treatment, the cells were harvested and re-plated into a trans-well. 24 h after incubation, the cells were fixed and the total and bottom cells are images to determine the migration activity. The quantitative results are shown in right panel. Mean ± SEM. *a*: significant compared with control group. *b*: significant compared with CuSO_4_ group. (**D**) The protein expression of EMT markers N-Cadherin and E-Cadherin were examined by Western blot. All experiments were performed three times and the representative images are shown.

To elucidate whether flavonoids could be developed as therapeutic agents for copper toxicity, drugs from different classes of flavonoids were examined including apigenin (Flavone), kaempferol (Flavonol), genkwanin (Flavone, Apigenin 7-methyl ether), and Fustin (Flavanonol). Cells treated with 1 μM, 2 μM, and 5 μM flavonoid drugs show no differences in cell viability between the untreated control (Figure 5A). The pretreatment of flavonoids inhibits copper-induced PINK1, LC3, and Snail expressions with various efficacy (Figure 5B). Moreover, flavonoids inhibit copper-induced cell migration (Figure 5C) and EMT marker expressions (Figure 5D). These results demonstrated that flavonoid is effective in reducing CuSO_4_-induced EMT through the inhibition of autophagy/mitophagy.

### 3.5. Association between Urinary Copper Levels and Lung Fibrotic Changes

The mean ± SD of age and BMI were 57.7 ± 11.2 years and 25.0 ± 3.8 Kg/m^2^. The ratio of male gender (32.6%), diabetes mellitus history (11.2%), hypertension history (27.6%), betel chewing (0.3%), alcohol consumption (16%), education levels (≤junior high school: 51.9%, senior high school: 30.1%, ≥college: 18.0%), air purifier use (84.5%) and lung fibrotic changes (36.2%) were determined for all participants. (Table S1). Participants with lung fibrotic changes were of older age (>60 years: 82.6% vs. 62.3%, *p* < 0.001), a higher ratio of hypertension (32.8% vs. 24.6%, *p* = 0.001), hyperlipidemia (3.4% vs. 1.2 %, *p* = 0.004) and air purifier use (88.8% vs. 82.0%, *p* = 0.001) (Table 1). In addition, individuals with lung fibrotic changes had significantly higher AST levels (IU/mL) (28.5 ± 11.7 vs. 27.8 ± 10.8, *p* < 0.001), higher fasting glucose levels (mg/dL) (101.2 ± 28.7 vs. 100.4 ± 27.4, *p* = 0.026), higher HbA1c levels (%) (5.93 ± 0.92 vs. 5.89 ± 0.98, *p* = 0.019) and lower platelet counts (256.5 ± 68.8 vs. 260.2 ± 68.6, *p* = 0.001) (Table 1). Individuals with lung fibrotic changes had borderline significantly higher urinary copper levels (µg/dL) (geometric mean: 1.45 vs. 1.39, *p* = 0.081) than non-lung fibrotic changes (Table 2). Urinary copper levels were significantly positively correlated with age (r = 0.17, *p* < 0.001), BMI (r = 0.11, *p* < 0.001), WBC (r = 0.11, *p* < 0.001), AST (r = 0.16, *p* < 0.001), ALT (r = 0.10, *p* < 0.001), serum creatinine (r = 0.13, *p* < 0.001), triglycerides (r = 0.13, *p* < 0.001), fasting glucose (r = 0.17, *p* < 0.001), HbA1c (r = 0.17, *p* < 0.001) and eosinophil counts (r = 0.10, *p* < 0.001). Urinary copper levels were significantly negatively correlated with platelet counts (r = −0.07, *p* = 0.010) and HDL-C (r = −0.14, *p* < 0.001) (Table 3). Subjects of male gender (mean ± SD, µg/dL) (male: 1.64 ± 0.89 vs. female: 1.57 ± 0.85, *p* = 0.014), older age (>60 years: 1.66 ± 0.92 vs. ≤60 years, *p* < 0.001), higher BMI (kg/m^2^) (>24: 1.65 ± 0.91 vs. ≤24: 1.52 ± 0.79, *p* < 0.001), lower education levels (≥college: 1.39 ± 0.57 vs. ≤junior high school: 1.68 ± 0.96, *p* < 0.001), diabetes mellitus (yes: 2.12 ± 1.23 vs. no: 1.53 ± 0.78, *p* < 0.001), hypertension (yes: 1.80 ± 1.07 vs. no: 1.51 ± 0.75, *p* < 0.001) and air purifier use (yes: 1.62 ± 0.88 vs.no: 1.47 ± 0.73, *p* = 0.016) had significantly higher urinary copper levels (Table S2). After adjustment for urinary creatinine, age, gender, BMI, AST, HbA1c, triglycerides, HDL-C, eosinophil counts, WBC, platelets, serum creatinine, educational levels, and air purifier use, we found that a unit increase in urinary copper concentration was significantly associated with an increased risk of lung fibrotic changes (odds ratio [OR] = 1.17, 95% confidence interval [CI] = 1.01–1.36, *p* = 0.038) (Table 4). 

## 4. Discussion

Copper is an essential trace element in the human body. The aberrant accumulation of copper is known to damage the liver and brain. However, the effects of copper on the lung are less addressed. In this study, we used the lung epithelial cells as an in vitro *model*, and also the association study between urine copper content with lung fibrosis, to evaluate the effects of copper on lung fibrosis. In humans, we found urinary copper levels significantly associated with lung fibrotic changes. Using a cell model, we demonstrated that copper activates a ROS-autophagy-EMT pathway in lung epithelial cells.

The generation of ROS is believed to be involved in metal-induced cell toxicity, including copper [20,21]. ROS act as signaling molecules to regulate autophagy through the activation of HIF-1α, Nrf2, and kinase signaling including mTOR and MAPKs [22]. The activation of autophagy by ROS was considered a protective mechanism for the clearance of damaged proteins and oxidative-induced cell death. However, excess autophagic targets may lead to cell death. Disregulated autophagy processes are believed to facilitate the development of several pulmonary diseases including lung fibrosis [23]. Furthermore, autophagy can regulate EMT and vice versa. Autophagy activation is believed to facilitate EMT through several mechanisms including providing survival signaling, secretion of invasive promoting enzymes such as MMP2, and degradation of E-Cadherin [24,25]. Moreover, copper exposure was demonstrated to induce autophagy via mTOR signaling [26]. Finally, targeting the ROS-autophagy pathway using flavonoids is effective to reduce copper-induced EMT. This result further supports the mechanism of copper-induced EMT changes, and also provides a possible therapeutic strategy for copper-induced pulmonary damage.

Copper signaling was essential for EMT induction. Knockdown *Ctr-1* (a transmembrane protein for copper uptake) expression inhibits CoCl_2_-induced EMT in breast cancer cells [27,28]. Moreover, Guo et al. demonstrated that the exposure of CuSO_4_ at 10~40 mg/kg intragastrically for three weeks induced EMT and fibrotic changes in mice lungs [29]. In addition, the more recent publication by Gaun et al. showed that mice exposed to 250 ppm CuSO_4_ for 16 weeks through drinking water lead to pulmonary damage including altered alveolar thickness and collagen deposition [30]. Furthermore, intranasal administration of copper oxide nanoparticles (CuO NP) induced lung fibrotic changes 28 days after administration [8]. Together, these studies provide in vivo evidence demonstrating the causal relationship between copper exposure and pulmonary fibrosis, and also indicate that EMT changes are the critical mechanistic event of copper-induced pulmonary fibrosis.

Mitochondria dysfunction is involved in the pathogenesis of pulmonary diseases such as chronic obstructive pulmonary disease (COPD), idiopathic pulmonary fibrosis (IPF), asthma, and lung cancer. Consistent with previous studies, we have also demonstrated that copper is a mitophagy-inducer in lung epithelial cells. Studies using the K562 cells, which are human lymphoblasts, have shown that a higher copper level leads to cell death by inducing mitochondria dysfunction. Nontoxic copper overload stimulates mitochondria turnover by mitophagy and mitochondria biogenesis [31]. Here we have observed a significant decrease in cell viability at the dosage of 100 μM CuSO_4_ (Figure 1). In a non-toxic dose of CuSO_4_, mitophagy/autophagy was induced rapidly. Besides, inhibition of autophagy blocked the copper-induced EMT. It is suggested that, in addition to stimulated mitochondria turnover, mitophagy is essential for the induction of EMT.

Several fibrotic markers were upregulated upon copper treatment by lung epithelial cells (Figure 2). Lysyl oxidase-like 2 (LOXL2) is a copper-dependent amine oxidase that plays an essential role in matrix remodeling and fibrogenesis. The expression level of LOXL2 was highly correlated with the severity of lung fibrosis, and was essential for the transition of fibroblast to myofibroblast through the TGF-beta/Smad pathway [32,33]. Blocking LOXL2 significantly attenuated fibrosis in a bleomycin-induced fibrotic model in mice [34]. Moreover, serum LOXL2 expression levels are associated with an increased risk for idiopathic pulmonary fibrosis disease progression in human studies [35]. LOXL2 up-regulation is associated with the metastasis of cancer such as colon cancer [36] and breast cancer [37], through regulating the EMT phenotype. In addition, LOXL2 can directly bind to Snail to suppress E-Cadherin expression [38]. In glioma, LOXL2 upregulation would trigger EMT through the induction of autophagy [39]. Here it is suggested that LOXL2, not only a fibrotic marker, is critical for mediating copper-induced fibrosis. 

The effects and mechanisms of copper on lung fibrosis were observed in cell and in rodent studies, however, evidence from human studies is still lacking. More recently, Jiag’s group has reported the positive correlation between urinary copper and the serum markers of chronic respiratory diseases (CRDs, including asthma, COPD, lung cancer, and emphysema), such as cytokines (IL-6, IL-8), and circRNAs in a case-control study (*n* = 101, 161; case vs. control). This study suggested that copper-exposure-induced inflammation and CRDs are mediated by circRNA, circ_0008882 [40]. The results support the damaging effects of copper on the pulmonary system. In this study, we performed a large-scale cohort study (*n* = 1458) to include the urinary copper level and LDCT-based lung fibrosis diagnostics to address this issue. Our results demonstrated that the increase in urinary copper levels increased the risk of pulmonary fibrosis (Table 1). 

Detoxification using a chelating agent is the most common therapeutic strategy for heavy metal toxicity. For copper poisoning, penicillamine is the most commonly used medication. However, the use of penicillin worsens the neurological condition and induces heavy adverse effects. The adjunctive antioxidant therapy with a chelation agent is reported to be useful for the treatment of metal toxicity [41]. The combination of antioxidants with chelating agents, such as Vitamin E, C, thiol group, zinc, and selenium, would reduce the dose of chelating agents and reduce the side effect. 

In this study, we have used the natural compound, flavonoid, as a treatment for copper-induced lung epithelial damage. Flavonoids are known to have metal-chelating properties to reduce metal-induced oxidative stress which can directly bind to iron and copper ions as an antioxidant [42]. Apigenin was reported to attenuate copper-mediated beta-amyloid neurotoxicity using a cell model of Alzheimer’s disease [43]. This study has shown that copper induced the production of ROS, reduced cellular levels of total-glutathione peroxidase (GSH), and superoxide dismutase (SOD), and caused cell death. Treatment of apigenin (10^−7^~10^−5^M) dose-dependently ameliorates copper-induced mitochondria membrane potential changes, cytochrome C release, and nuclear apoptosis. In this study, various flavonoids including flavones (apigenin, kaempferol), o-methylated flavone (genkwanin), and flavanonol (fustin) were shown to reverse copper-induced EMT changes in lung epithelial cells. Our results provide evidence that flavonoids, especially kaempferol, may be another effective therapeutic agent for copper-induced lung fibrosis.

Many studies reveal that the incidence and mortality of idiopathic pulmonary fibrosis is increasing worldwide over the last two decades [44]. However, whether the increase in pulmonary fibrosis is caused by idiopathy itself or is due to the global increase in secondary causes of lung fibrosis remains unknown. Previous systematic overview studies revealed that environmental metal exposure was associated with interstitial pulmonary fibrosis [45]. However, the potential role of copper exposure in fibrotic interstitial lung disease is not well established in humans [45]. The significant association of copper exposure with lung fibrotic changes that are linked to mitophagy and EMT of lung epithelial cells in our study provides contributions for future studies in interstitial lung disease and idiopathic pulmonary fibrosis.

## 5. Conclusions

In this study, we applied both experimental and epidemiology methods and demonstrate the effects and novel mechanisms of copper exposure on lung fibrosis. Moreover, using the cell model as a drug screening platform, we demonstrated the beneficial role of flavonoids on copper-induced pulmonary fibrotic changes to provide therapeutic insights. The therapeutic effects of flavonoids are worth further validation using animal models and human studies.

## Figures and Tables

**Table 1:** Demographic characteristics and blood biochemistry levels of participants between lung fibrotic and non-lung fibrotic changes (*n* = 1458).

Characteristics	Non-Lung Fibrotic (*n* = 930)	Lung Fibrotic (*n* = 528)	*p*
Category variable, *n* (%)			
Gender			0.383
Female	619 (66.6)	364 (68.9)	
Male	311 (33.4)	164 (31.1)	
Age (yrs)			<0.001
≤60	351 (37.7)	92 (17.4)	
>60	579 (62.3)	436 (82.6)	
BMI (kg/m^2^)			0.543
≤24	379 (40.8)	224 (42.4)	
>24	551 (59.2)	304 (57.6)	
Education levels			<0.001
≤junior high school	431 (46.3)	325 (61.6)	
Senior high school	297 (31.9)	142 (26.9)	
≥college	202 (21.7)	61 (11.6)	
Diabetes mellitus			0.344
No	831 (89.4)	463 (87.7)	
Yes	99 (10.6)	65 (12.3)	
Hypertension			0.001
No	701 (75.4)	355 (67.2)	
Yes	229 (24.6)	173 (32.8)	
Hyperlipidemia			0.004
No	919 (98.8)	510 (96.6)	
Yes	11 (1.2)	18 (3.4)	
Alcohol consumption			0.882
No	780 (83.9)	445 (84.3)	
Yes	150 (16.1)	83 (15.7)	
Physical activity ^a^			0.083
No	234 (25.2)	111 (21.0)	
Yes	696 (74.8)	417 (79.0)	
Air purifier			0.001
No	167 (18.0)	59 (11.2)	
Yes	763 (82.0)	469 (88.8)	
Serum, continuous variable, mean ± SD (range)			
WBC ×10^3^ (/μL)	5.9 ± 1.5 (2.4, 14.0)	5.8 ± 1.5 (2.5, 12.2)	0.226
Platelet ×10^3^ (/μL)	260.2 ± 68.6 (60.0, 794.0)	256.5 ± 68.8 (117.0, 672.0)	0.001
AST (IU/mL)	27.8 ± 10.8 (12.0, 102.0)	28.5 ± 11.7 (14.0, 177.0)	<0.001
ALT (IU/mL)	25.9 ± 18.7 (3.0, 190.0)	25.8 ± 18.5 (4.0, 185.0)	0.852
Creatinine (mg/dL)	0.9 ± 0.3 (0.5, 5.0)	0.9 ± 0.3 (0.6, 6.8)	0.071
Cholesterol (mg/dL)	200.3 ± 36.5 (91.0, 420.0)	200.6 ± 36.8 (100.0, 386.0)	0.912
Triglycerides (mg/dL)	121.2 ± 80.3 (25.0, 1129.0)	121.5 ± 79.6 (29.0, 952.0)	0.705
HDL-C (mg/dL)	53.6 ± 13.6 (25.0, 100.2)	53.5 ± 13.5 (24.4, 94.3)	0.917
LDL-C (mg/dL)	119.6 ± 32.7 (25.0, 306.0)	119.6 ± 33.0 (34.0, 276.0)	0.978
Fasting glucose (mg/dL)	100.4 ± 27.4 (57.0, 325.0)	101.2 ± 28.7 (72.0, 348.0)	0.026
HbA1c (%)	5.89 ± 0.98 (4.20, 12.30)	5.93 ± 0.92 (4.20, 12.90)	0.019
Eosinophil (/μL)	134.6 ± 124.3 (0.1, 1010.0)	127.6 ± 128.9 (0.1, 1070.0)	0.090
IgE (IU/mL)	76.5 ± 235.1 (0.1, 4439.4)	115.2 ± 518.5 (0.1, 7618.3)	0.128

^a^ Do you undertake at least 150 min per week of moderate-intensity aerobic activity or 75 min per week of vigorous aerobic activity or an equivalent combination? Normal range (AST < 42 IU/mL, ALT < 40 IU/mL, cholesterol < 200 mg/dL, triglycerides < 150 mg/dL, HDL-C > 40 mg/dL, LDL-C < 130 mg/dL, fasting glucose < 100 mg/dL, HbA1c < 6%, eosinophil count < 400/μL and IgE < 87 IU/mL).

**Table 2:** Urinary copper and creatinine levels between individuals with lung fibrotic and non-lung fibrotic changes (*n* = 1458).

Item	Mean	SD	GM	Min.	25th	Median	75th	Max.	*p*
Urine									
Copper (µg/dL)									0.081
Non-lung fibrotic	1.55	0.78	1.39	0.05	1.05	1.43	1.86	8.25	
Lung fibrotic	1.69	0.99	1.45	0.05	1.11	1.48	1.90	9.94	
Creatinine (mg/dL)									0.107
Non-lung fibrotic	130.13	59.31	115.92	30.20	83.60	124.55	171.83	296.60	
Lung fibrotic	124.68	56.85	111.25	30.40	80.63	116.70	160.95	297.50	

**Table 3:** Spearman correlation coefficient of urinary copper levels, age, BMI, and serum biochemistry levels (*n* = 1458).

Items	Copper	Age	BMI	WBC	PLT	AST	ALT	Cr	CHOL	TG	HDL-C	LDL-C	Glucose	HbA1c	Eosinophil	IgE
Copper	1															
Age	0.17 ^**^	1														
BMI	0.11 ^**^	0.13 ^**^	1													
WBC	0.11 ^**^	−0.05 ^*^	0.25 ^**^	1												
PLT	0.07 ^*^	−0.34 ^**^	0.01	0.30 ^**^	1											
AST	0.16 ^**^	0.26 ^**^	0.17 ^**^	0.06 ^*^	−0.16 ^**^	1										
ALT	0.10 ^**^	0.11 ^**^	0.35 ^**^	0.14 ^**^	−0.13 ^**^	0.68 ^**^	1									
Cr	0.13 ^**^	0.25 ^**^	0.13 ^**^	0.09 ^**^	−0.21 ^**^	0.15 ^**^	0.12 ^**^	1								
CHOL	−0.04	0.03	−0.03	0.01	0.10 ^**^	0.01	0.01	−0.06 ^*^	1							
TG	0.13 ^**^	0.14 ^**^	0.33 ^**^	0.27 ^**^	0.07 ^**^	0.14 ^**^	0.27 ^**^	0.13 ^**^	0.18 ^**^	1						
HDL-C	−0.14 ^**^	−0.12 ^**^	−0.31 ^**^	−0.20 ^**^	0.06 ^*^	−0.05 ^*^	−0.19 ^**^	−0.26 ^**^	0.34 ^**^	−0.51 ^**^	1					
LDL-C	−0.003	0.01	0.07 ^*^	0.07 ^*^	0.09 ^**^	−0.003	0.05	−0.01	0.84 ^**^	0. 15 ^**^	0.04	1				
Glucose	0.17 ^**^	0.34 ^**^	0.32 ^**^	0.15 ^**^	−0.09 ^**^	0.16 ^**^	0.22 ^**^	0.11 ^**^	−0.02	0.26 ^**^	−0.22 ^**^	0.01	1			
HbA1c	0.17 ^**^	0.34 ^**^	0.29 ^**^	0.22 ^**^	−0.02	0.19 ^**^	0.19 ^**^	0.08 ^**^	−0.01	0.28 ^**^	−0.23 ^**^	0.01	0.63 ^**^	1		
Eosinophil	0.10 ^**^	−0.07 ^**^	0.10 ^**^	0.24 ^**^	0.08 ^**^	0.08 ^**^	0.06 ^*^	0.13 ^**^	0.001	0.12 ^**^	−0.11 ^**^	0.04	0.03	0.13 ^**^	1	
IgE	0.04	0.01	0.09 ^**^	0.06 ^*^	0.01	−0.02	0.03	0.08 ^**^	−0.08 ^**^	−0.01	−0.06 ^*^	−0.04	0.05	0.05	0.13 ^**^	1

Abbreviations: PLT: Platelet count, AST: Aspartate aminotransferase, ALT: Alanine aminotransferase, Cr: Creatinine, TG: triglycerides. ^*^ *p* < 0.05, ^**^ *p* < 0.01.

**Table 4:** Odds ratio (95% Confidence Interval) for lung fibrotic changes associated with a unit increase of urinary copper concentration (*n* = 1458).

Urinary Copper (ug/dL)	OR	95% CI	*p*
Lung fibrotic change			
Model 1	1.27	(1.10, 1.46)	0.001
Model 2	1.17	(1.01, 1.35)	0.031
Model 3	1.19	(1.03, 1.39)	0.021
Model 4	1.17	(1.01, 1.36)	0.038

Model 1: adjusted for urinary creatinine. Model 2: adjusted for Model 1 in addition to age, gender, and BMI. Model 3: adjusted for Model 2 in addition to AST, HbA1c, triglycerides, HDL-C, eosinophil counts, WBC, platelets, and serum creatinine. Model 4: adjusted for Model 3 in addition to educational levels and air purifier use.

## Data Availability

The data presented in this study are available on request from the corresponding author. The data are not publicly available due to privacy.

## Supplementary Materials

The following supporting information can be downloaded at: https://www.mdpi.com/article/10.3390/antiox12020532/s1, Table S1: Demographic characteristics and lung fibrotic changes of all participants (*n* = 1458); Table S2: Comparison of urinary copper levels between demographic characteristics (*n* = 1458).
