# Supplementary material for: Copper Exposure Induces Epithelial-Mesenchymal Transition-Related Fibrotic Change via Autophagy and Increase Risk of Lung Fibrosis in Human
Source: Antioxidants (Basel). 2023 Feb 20;12(2):532. doi: 10.3390/antiox12020532 (PMC9952124; doi:10.3390/antiox12020532)
Supplement: Supplementary file 1 [file antioxidants-12-00532-s001.zip › antioxidants-2188977-supplementary.pdf]

Supplementary materials

Table S1 Demographic characteristics and lung fibrotic changes of all participants (n= 1458)

Table S2 Comparison of urinary copper levels between demographic characteristics (n= 1458)

Table S1 Demographic characteristics and lung fibrotic changes of all participants (n= 1458)

Characteristics	All participants
Continuous variable, mean ±SD (range)	
Age (yrs.)	57.7±11.2(39.0,84.0)
BMI (Kg/m^2)	25.0±3.8(14.3,43.7)
Waist circumference (cm)	83.0±10.4(43.0,128.0)
Systolic blood pressure (mmHg)	132.5±20.3(77.0,235.0)
Diastolic blood pressure (mmHg)	77.2±11.5(45.0,136.0)
Category variable; n (%)	
Gender	
Female	983(67.4)
Male	475(32.6)
Diabetes mellitus history	
No	1294(88.8)
Yes	164(11.2)
Hypertension history	
No	1056(72.4)
Yes	402(27.6)
Betel chewing	
No	1454(99.7)
Yes	4(0.3)
Alcohol consumption	
No	1225(84.0)
Yes	233(16.0)
Education	
≤junior high school	756(51.9)
Senior high school	439(30.1)
≥college	263(18.0)
Physical activity[a]	
No	345(23.7)
Yes	1113(76.3)
Air purifier	
No	226(15.5)
Yes	1232(84.5)
Lung fibrotic change	
No	930(63.8)
Yes	528(36.2)

[a]Do you undertake at least 150 min per week of moderate-intensity aerobic activity or 75 min per week of vigorous aerobic activity or an equivalent combination?

Table S2 Comparison of urinary copper levels between demographic characteristics (n= 1458)

Characteristics	Urinary copper, μg/dL	p
Mean±SD (range)		
Gender		0.014
Female	1.57±0.85(0.05,9.94)	
Male	1.64±0.89(0.20,7.24)	
Age (yrs)		<0.001
≤60	1.44±0.68(0.05,7.00)	
>60	1.66±0.92(0.05,9.94)	
BMI (kg/m2)		<0.001
≤24	1.52±0.79(0.05,7.00)	
>24	1.65±0.91(0.05,9.94)	
Education		<0.001
≤junior high school	1.68±0.96(0.05,9.94)	
Senior high school	1.57±0.80(0.05,7.15)	
≥college	1.39±0.57(0.20,3.87)	
Diabetes mellitus history		<0.001
No	1.53±0.78(0.05,8.25)	
Yes	2.12±1.23(0.34,9.94)	
Hypertension history		<0.001
No	1.51±0.75(0.05,7.00)	
Yes	1.80±1.07(0.05,9.94)	
Hyperlipidemia		0.446
No	1.59±0.86(0.05,9.94)	
Yes	1.66±0.85(0.38,4.59)	
Alcohol consumption		0.718
No	1.60±0.87(0.05,9.94)	
Yes	1.58±0.83(0.30,7.24)	
Physical activity		0.506
No	1.55±0.75(0.37,7.00)	
Yes	1.61±0.89(0.05,9.94)	
Air purifier		0.016
No	1.47±0.73(0.05,4.54)	
Yes	1.62±0.88(0.05,9.94)	
